# Improved correspondence of fMRI visual field localizer data after cortex-based macroanatomical alignment

**DOI:** 10.1038/s41598-022-17909-2

**Published:** 2022-08-22

**Authors:** Mishal Qubad, Catherine V. Barnes-Scheufler, Michael Schaum, Eva Raspor, Lara Rösler, Benjamin Peters, Carmen Schiweck, Rainer Goebel, Andreas Reif, Robert A. Bittner

**Affiliations:** 1 Department of Psychiatry, Psychosomatic Medicine and Psychotherapy and Brain Imaging Center, University Hospital Frankfurt, Goethe University, Frankfurt am Main, Germany; 2 Leibniz Institute for Resilience Research, Mainz, Germany; 3 Netherlands Institute for Neuroscience, Amsterdam, The Netherlands; 4 Institute of Medical Psychology, University Hospital Frankfurt, Goethe University, Frankfurt am Main, Germany; 5 Zuckerman Mind Brain Behavior Institute, Columbia University, New York, NY USA; 6 Department of Cognitive Neuroscience, Faculty of Psychology and Neuroscience, Maastricht University, Maastricht, The Netherlands; 7 Ernst Strüngmann Institute for Neuroscience (ESI) in Cooperation With Max Planck Society, Frankfurt am Main, Germany

**Keywords:** Attention, Perception, Visual system

## Abstract

Studying the visual system with fMRI often requires using localizer paradigms to define regions of interest (ROIs). However, the considerable interindividual variability of the cerebral cortex represents a crucial confound for group-level analyses. Cortex-based alignment (CBA) techniques reliably reduce interindividual macroanatomical variability. Yet, their utility has not been assessed for visual field localizer paradigms, which map specific parts of the visual field within retinotopically organized visual areas. We evaluated CBA for an attention-enhanced visual field localizer, mapping homologous parts of each visual quadrant in 50 participants. We compared CBA with volume-based alignment and a surface-based analysis, which did not include macroanatomical alignment. CBA led to the strongest increase in the probability of activation overlap (up to 86%). At the group level, CBA led to the most consistent increase in ROI size while preserving vertical ROI symmetry. Overall, our results indicate that in addition to the increased signal-to-noise ratio of a surface-based analysis, macroanatomical alignment considerably improves statistical power. These findings confirm and extend the utility of CBA for the study of the visual system in the context of group analyses. CBA should be particularly relevant when studying neuropsychiatric disorders with abnormally increased interindividual macroanatomical variability.

## Introduction

The visual system includes a multitude of topographical representations of varying resolution across increasingly specialized visual areas^1^. Functional magnetic resonance imaging (fMRI) offers a variety of methods either to map these topographical representations in full, or to localize specific visual areas or retinotopic positions within their topography. These approaches are essential not only for the fine-grained study of fundamental properties of the visual system^1^, but also for investigating the role of these areas for higher-order cognitive processes such as visual attention and working memory^2–6^. This also extends to translational studies of visual dysfunction and its cognitive consequences in neuropsychiatric disorders^7,8^.

Methods for fMRI-based visual mapping, i.e., techniques to define regions of interest in the visual system based on specific functional properties, fall in in three broad categories: retinotopic mapping, visual field localizer and functional localizer paradigms. Retinotopic mapping and the more advanced population receptive field (pRF) mapping allow the complete delineation of early visual areas^1,9,10^. Conversely, visual field localizer paradigms can map a circumscribed region within a retinotopically organized visual area^11,12^. Finally, functional localizers can detect higher-order visual areas such as the fusiform face area (FFA), parahippocampal place area (PPA), extrastriate body area and lateral occipital complex (LOC), which are clustered and show specialization for the processing of specific categories of complex visual information^1,13,14^. In most fMRI studies, high interindividual anatomical variability of cortical areas in terms of both size and location constitutes an important challenge^15–23^. For instance, it has been shown that primary visual cortex (V1) can differ in size by about twofold between individuals^17^. Furthermore, anatomical variability in terms of location has been shown to be particularly pronounced in extrastriate﻿ visual areas^24^. This crucial confound reduces the power to reliably map visual areas at the group level.

One way to mitigate this problem is to pool single-subject regions of interest (ROIs), while simultaneously using the overall group-based probability for that ROI at each point in a Cartesian coordinate system as a constraint^25–27^. While such a single-subject-based analysis improves sensitivity and functional resolution compared to a standard group-based approach, it does not actually reduce macroanatomical variability. Additionally, studying the interplay between visual areas and other cortical areas more directly involved in higher-order cognitive processes with whole-brain methods such as functional connectomics network analyses^28^ might preclude a single-subject based strategy.

Group-based analyses typically require spatial normalization of structural and functional imaging data to a common Cartesian coordinate system such as Talairach^29^ or MNI^30^ space. In its most basic form, volume-based spatial normalization employs a linear transformation that matches the overall extent of the brains to a standard brain template. While transformation into Talairach space relies on anatomical landmarks, transformation into MNI space utilizes fully data-driven registration of structural images to an average template brain^30^. While these spatial normalization approaches inherently result in an alignment of brains, the underlying algorithms are not optimized specifically for aligning homologous brain structures. Conversely, more refined methods employ non-linear warping algorithms guided by intensity differences to improve macroanatomical alignment^31^. Thus, all of these methods can be categorized as volume-based alignment (VBA) techniques. However, both linear and nonlinear VBA mostly disregard the topological properties of the cerebral cortex and its geometric features such as sulci and gyri. Consequently, VBA methods result in a considerable amount of residual interindividual anatomical variability^32,33^.

Surface-based procedures constitute an important alternative approach. Surface-based spatial normalization typically uses a geodesic coordinate system, which allows for a two-dimensional representation of the cerebral cortex and respects the cortical topography to a much larger degree than traditional Cartesian coordinate systems^18,34^. This approach offers two main advantages over VBA. First, surface-based spatial normalization allows to constrain data readout and data pre-processing such as spatial smoothing to cortical tissue. This reduces signal contamination by white matter and cerebrospinal fluid substantially and also mostly precludes contamination from cortical areas proximal in volume space but considerably more distant in surface space. Overall, this approach enhances the signal-to-noise ratio (SNR). Consequently, spatial smoothing in surface space is superior to spatial smoothing in volume space^19,35^. The second advantage of surface-based spatial normalization is the possibility to use individual cortical folding patterns for an additional, fully data-driven macroanatomical alignment of the cerebral cortex^34^. Compared to VBA techniques, these cortex-based alignment (CBA) methods considerably improve anatomical correspondence of cortical structures while respecting cytoarchitectonic boundaries^36^. Thus, CBA leads to a notable reduction of interindividual anatomical variability^18,34,37–39^.

Importantly, previous studies have often exclusively compared surface-based data before and after macroanatomical alignment^19,40^, essentially using the former approach as a proxy for VBA. Yet, this comparison only reflects the second advantage of CBA, namely the use of macroanatomical alignment instead of VBA. However, in this case both data sets benefit equally from reduced signal contamination, likely underestimating the full effects of CBA. Assessing the impact of this first advantage of surface-based analyses in isolation requires a comparison of VBA with a surface-based analysis without macroanatomical alignment. We refer to this intermediate approach as a “surface-based analysis using VBA” (SBAV). Thus, assessing both benefits of CBA requires the comparison of three approaches: VBA, SBAV and CBA.

Due to the advantageous properties outlined above, CBA methods have been proposed as an alternative approach to VBA specifically for the visual system^26^. Several studies have compared the impact of VBA and CBA methods on specific visual mapping techniques. For retinotopic mapping, an improvement of functional overlap in both V1 and V2 after CBA has been demonstrated^34,41^. For functional localizer data, CBA substantially increases the overlap of object processing areas LOC, FFA and PPA across subjects^19,42–44^. Conversely, the effects of CBA on visual field localizer paradigms mapping specific retinotopic positions have not been studied. Thus, the utility of CBA has been demonstrated for two of the three main categories of visual mapping methods, i.e., those methods, which map whole areas, either defined primarily by cytoarchitectonic (e.g. V1) or functional (e.g. FFA) properties. Conversely, it remains unclear, to which degree CBA can improve the alignment of ROIs mapped by visual field localizer paradigms. Such paradigms are required for the detailed study of the local processing of simple visual stimuli in early visual areas^11,12,45–47^. Flashing checkerboards covering the exact area of interest within the visual field are primarily used for this purpose. Checkerboards lead to a particularly strong BOLD-signal increase in early visual areas (V1–V3)^48^. To maximize fidelity of the resulting localizer maps, visual field localizer paradigms typically utilize the fact that attentional modulation induced by task demands significantly enhances response reliability across visual areas. This can be achieved by adding a simple target-detection task^49^.

We used such an attention-enhanced visual field localizer paradigm to map a circumscribed location in each visual quadrant across early visual areas aiming to define ROIs suitable for the study of higher cognitive processes. We chose a CBA method using a dynamic group average as the target brain^19^. Thus, we eliminated the possible confound of a static CBA target based on an individual brain, whose cortical folding pattern might by chance deviate considerably from the group average.

Our primary goal was to examine the effects of CBA for a visual field localizer paradigm. More specifically, we aimed to determine, whether macroanatomical alignment improves the reliability of mapping subregions within retinotopically organized visual areas delineated by such a paradigm at the group level. To this end, in addition to the analysis of the full single-subject ROIs, we also examined the correspondence of single-subject ROI peak vertices, i.e., single vertices showing the strongest level of activation in each subject for each visual quadrant. We conducted this analysis, because peak vertices are a good approximation of the center of a ROI and thus allow for a more precise assessment and visualization of the effects of macroanatomical alignment. Based on previous findings for other localizer paradigm classes and the relatively good structural–functional correspondence in posterior occipital cortex, we expected to observe a benefit of CBA compared to SBAV when aligning subregions within early visual cortex for both full ROIs and peak vertices.

Our second goal was to examine the effects of SBAV. More specifically, we aimed to assess the impact of surface-based functional data readout and pre-processing without macroanatomical alignment. Here, we expected a general improvement of the SNR for SBAV compared to VBA and a corresponding global increase in group ROI size for all visual quadrants. Notably, several studies have shown differential response properties such as receptive field size by visual quadrant or hemifield for homologous early visual areas. For instance, previous studies reported improved behavioral performance and higher BOLD-signal amplitudes in the lower visual hemifield^50–53^. We were therefore also interested, whether we could observe differences between upper and lower visual hemifields in our group analysis after CBA.

Overall, the aim of the study was to close an important gap in the evaluation of CBA for the study of the visual system. Since visual field localizers are crucial for investigating contributions of the visual system to higher-order cognitive processes, our results should have implications for the study of visual cognition in both basic and translational neuroscience research.

## Results

### Visual quadrant ROIs (group level)

**Figure 1:**
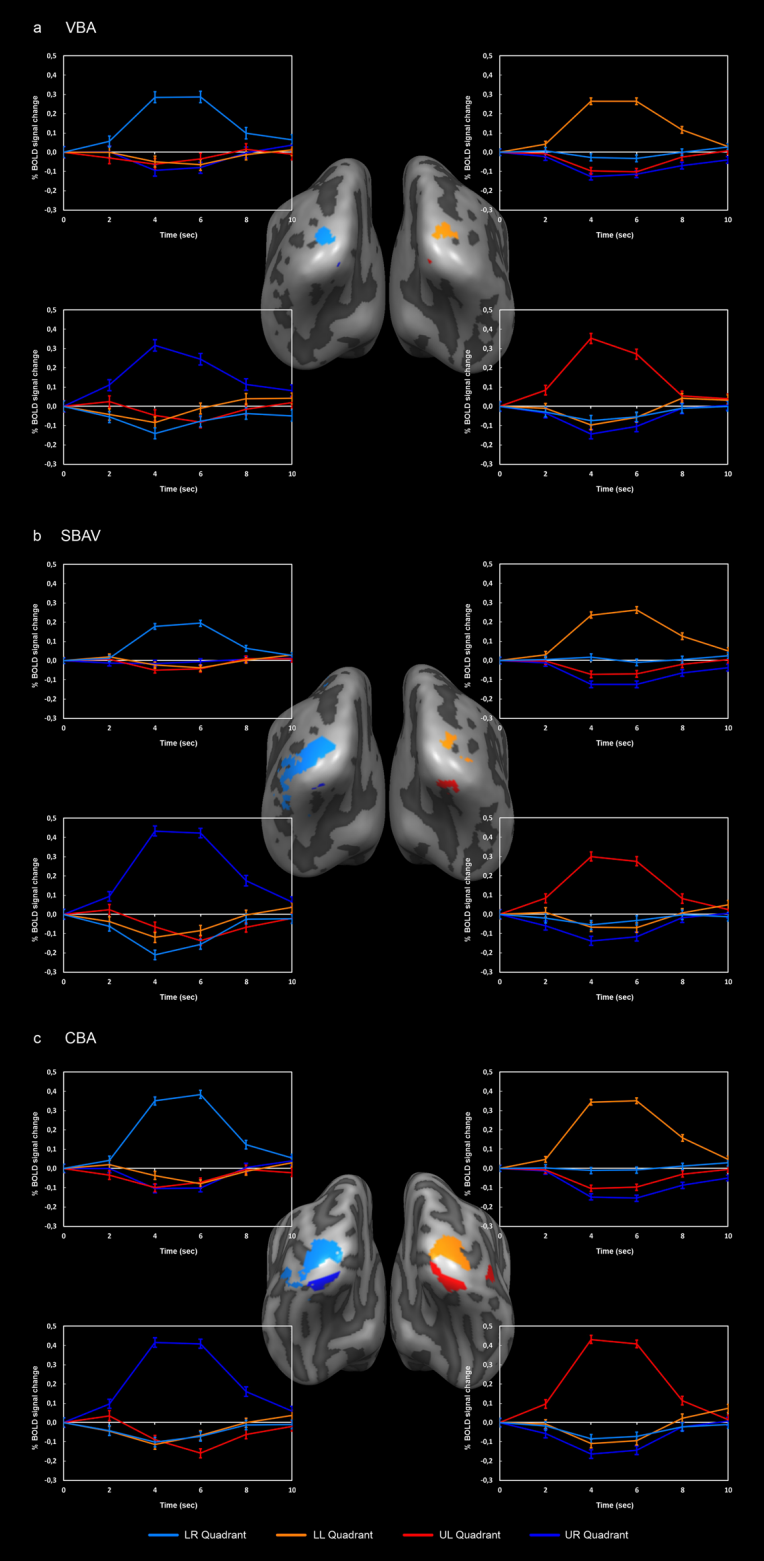
Group analysis of visual quadrants. (**a**) VBA results. Maps and average timecourses were computed in volume space; maps were projected on the non-aligned average surface representation. (**b**) SBAV results. Maps and average timecourses were computed in surface space; maps were projected on the non-aligned surface representation. (**c**) CBA results. Maps and average timecourses were computed in surface space; maps were projected on the aligned average surface representation. Overall, two out of four visual quadrant ROIs exhibited a pattern of continuously increasing cluster size, reflecting an increasing extent of significant position selectivity across alignment techniques. Additionally, while ROI size for the lower left visual quadrant decreased slightly from VBA to SBAV, ROI size for CBA was also by far the largest. Only the ROI of the lower right visual quadrant showed a cluster size decrease after CBA. Average timecourses (incl. standard error of the mean) showed clear position selectivity with a strong BOLD signal increase for the position of interest and no BOLD signal increase for the other three positions. ROI/graph colors: light-blue = lower right (LR) visual quadrant, orange = lower left (LL) visual quadrant, red = upper left (UL) visual quadrant, dark-blue = upper right (UR) visual quadrant.

Group-level mapping of the four visual quadrants revealed notable differences for the three alignment techniques (VBA, SBAV, CBA) (Fig. 1, Tables 1, 2). For the lower right visual quadrant, ROI size increased considerably from VBA to SBAV, but decreased for CBA (Table 2). For the lower left visual quadrant, ROI size decreased slightly from VBA to SBAV, but increased considerably for CBA. For the upper left visual quadrant, ROI size increased considerably from VBA to SBAV and increased further for CBA. For the upper right visual quadrant, ROI size increased slightly from VBA to SBAV and increased considerably for CBA. Thus, two out of four visual quadrant ROIs exhibited a pattern of continuously increasing cluster size, reflecting an expansion of significant position selectivity across alignment techniques. Additionally, while ROI size for the lower left visual quadrant decreased slightly from VBA to SBAV, ROI size for CBA was also by far the largest. Furthermore, while ROI size decreased for the lower right visual quadrant after CBA, for SBAV this ROI showed by far the greatest extent of any ROI for any alignment technique, even encompassing posterior parts of temporal cortex.

**Table 1:** Group ROIs.

Region of interest	Analysis method	Number of vertices	Number of voxels	TAL coordinates		
				**x**	**y**	**z**
Lower right visual quadrant	VBA	47	953	− 22	− 90	2
	SBAV	295	NA	− 42	− 64	5
	CBA	161	NA	− 24	− 91	− 4
Lower left visual quadrant	VBA	46	828	19	− 94	2
	SBAV	28	NA	23	− 90	3
	CBA	127	NA	9	− 94	− 5
Upper left visual quadrant	VBA	4	55	4	− 87	− 9
	SBAV	47	NA	24	− 75	− 14
	CBA	82	NA	18	− 77	− 15
Upper right visual quadrant	VBA	3	1	− 5	− 88	− 13
	SBAV	6	NA	− 18	− 80	− 15
	CBA	58	NA	− 18	− 80	− 15

Size and Talairach coordinates of the group ROIs of the corresponding visual quadrants for the VBA, SBAV and CBA data sets.

For ROI size comparison, we focused exclusively on the number of vertices. Importantly, we only provide the number of voxels of each VBA ROI as a reference to ensure a comprehensive reporting of our findings. In three out of four visual quadrant ROIs we observed the largest cluster size for CBA, which is indicative of the highest degree of position selectivity for the most advanced alignment technique.

**Table 2:** Changes of group ROI size compared between alignment methods.

	VBA → SBAV (%)	VBA → CBA (%)	SBAV → CBA (%)
Lower right visual quadrant	528	243	− 45
Lower left visual quadrant	− 39	176	354
Upper left visual quadrant	1075	1950	74
Upper right visual quadrant	100	833	867

We used the following formula: {(size_ROI_*Quad*_[AM_*m*_] − size_ROI_*Quad*_[AM_*n*_])/size_ROI_*Quad*_[AM_*n*_]} × 100. For SBAV compared to VBA, we observed a group ROI size increase in three out of four visual quadrants. For CBA compared to VBA, we observed a group ROI size increase in all four visual quadrants. For CBA compared to SBAV we observed a group ROI size increase in three out of four visual quadrants. *Quad* visual quadrant of interest, *AM* alignment method (VBA, SBAV, CBA). n and m specify AMs, with m referring to the less advanced AM and n referring to the comparatively more advanced AM.

Within group ROIs, average time courses showed clear position selectivity, which was not further affected by alignment technique as indicated by the negative results of our linear mixed models (Table 3). Notably, asymmetry indices (AIs) revealed markedly greater vertical symmetry of both upper and lower hemifield ROIs for VBA and CBA compared to SBAV (Table 4). After CBA, ROI sizes for the lower visual hemifield were considerably larger than for the upper visual hemifield (Table 1).

**Table 3:** Effect of alignment method on position selectivity within group ROIs.

visual quadrant	Sum Sq	Mean Sq	NumDF	DenDf	F value	p value	p value (corr.)
Lower right visual quadrant	183.2	92	2	147*	0.31	0.737	1.000
Lower left visual quadrant	2660.2	1330.1	2	98	1.25	0.291	1.000
Upper right visual quadrant	2546.2	1273.1	2	98	3.36	0.039	0.156
Upper left visual quadrant	73678	36839	2	98	1.19	0.308	1.000

To test whether the strength of position selectivity within corresponding group ROIs across alignment techniques increases for the more advanced alignment techniques, we conducted separate linear mixed models with random intercept for each visual quadrant. For each position, we used each subject’s t-values as the dependent variable and the alignment techniques (VBA, SBAV and CBA) as the independent variable. We adjusted p-values using Bonferroni correction. We did not observe any significant effect (all p adjusted > 0.05), indicating that this measure of position selectivity within corresponding group ROIs was not affected by alignment technique. Thus, while for each alignment method and visual quadrant the corresponding ROI did show significant position selectivity, the strength of within-ROI position selectivity did not increase for more advanced alignment techniques.

*Random effect variance estimate at the subject-level for the LR ROI was 0, resulting in a singular fit when using lmer. Therefore, results for LR were estimated without random intercept are thus equivalent to a regular ANOVA.

*Sum Sq* sum of squares, *Mean Sq* mean square, *NumDf* degrees of freedom in the numerator, *DenDf* degrees of freedom in the denominator.

**Table 4:** Vertical and horizontal asymmetry indices (AIs).

Symmetry	ROI comparison	AI (VBA)	AI (SBAV)	AI (CBA)
Vertical	LR and LL	1.1	82.7	11.8
	UL and UR	14.3	77.4	17.1
Horizontal	LR and UR	88.0	96.0	47.0
	LL and UL	84.0	25.3	21.5

To assess the impact of the three alignment techniques on horizontal and vertical symmetry of our group-level ROIs, we computed a ROI size AI between each pair of ROIs. AIs revealed greater vertical symmetry of the upper and lower hemifield ROIs for VBA and CBA compared to SBAV.

*LR* lower right visual quadrant, *LL* lower left visual quadrant, *UL* upper left visual quadrant, *UR* upper right visual quadrant.

### Probability maps

**Figure 2:**
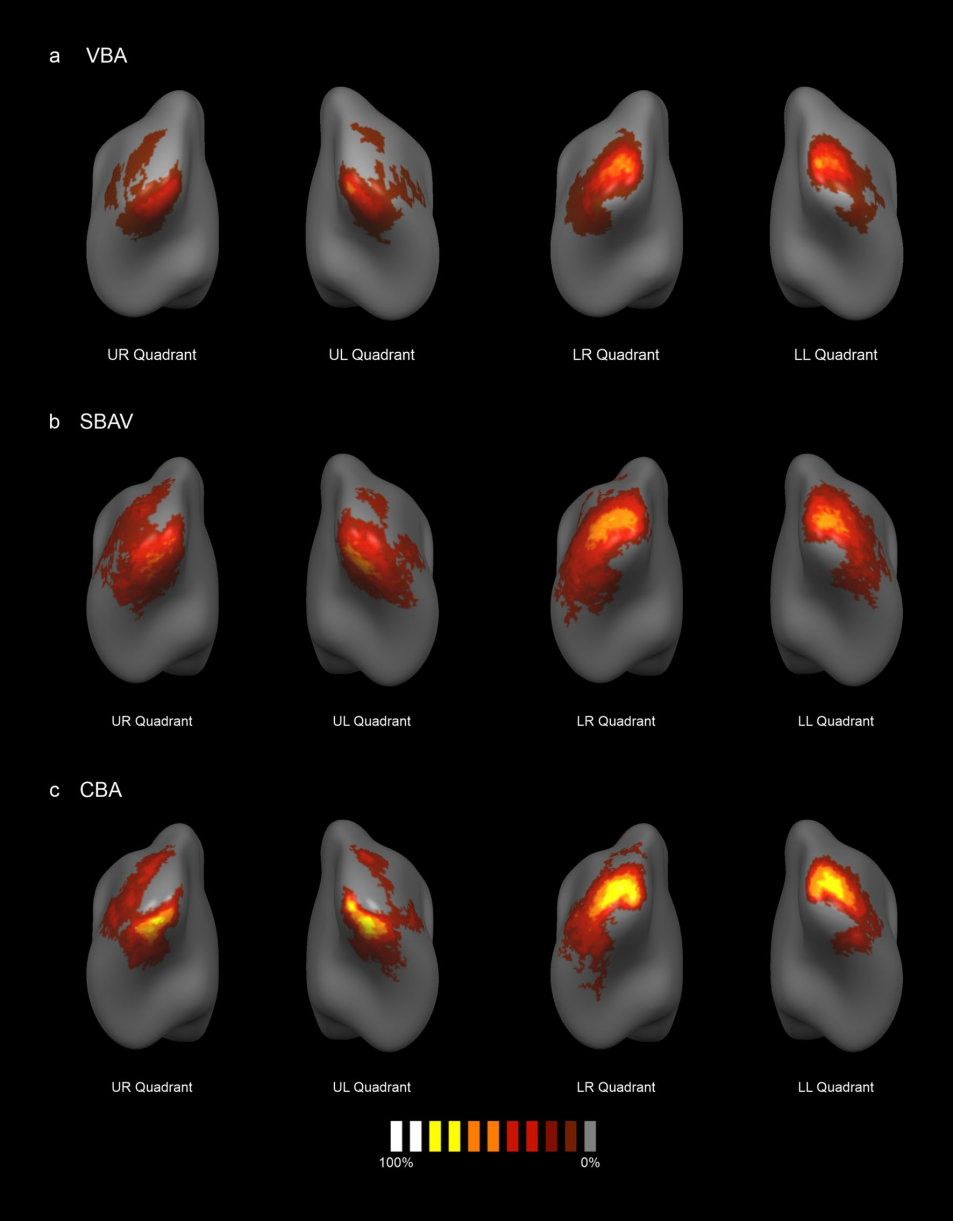
Probability maps (PMs). PMs indicating the probability of activation overlap across subjects for each visual quadrant. The color code gray-to-white indicates the probability of activation overlap of single-subject maps, thresholded at a minimum of 10% probability of activation overlap. Single-subject maps were thresholded at p < 0.05 (uncorr.). We also applied a cluster level threshold of 100 vertices. (**a**) PMs for VBA showed a maximum probability of activation overlap of up to 55%. (**b**) PMs for SBAV showed a maximum probability of activation overlap of up to 66%. (**c**) PMs for CBA showed a maximum probability of activation overlap of up to 86%.

For all three data sets, the maximum probability of activation overlap was consistently located at the center of each ROI as defined in our previous group analysis (Fig. 1, Tables 1, 5). For VBA data, probability maps (PMs) showed a relatively wide spread of functional activation around the core ROIs (Fig. 2a, Table 5). Maximum probability of activation overlap was 55%. For SBAV data, PMs showed an even wider spread of functional activation around the core ROIs (Fig. 2b, Table 5). Maximum probability of activation overlap was 66%. For CBA data, PMs showed a noticeable decrease in the spread of functional activation around the core ROIs with a corresponding increase in the maximum probability of overlap at the center of the core ROIs (Fig. 2c, Table 5). Maximum probability of activation overlap was 86%.

**Table 5:** Extent of probability maps.

Probability map	Analysis method	Number of vertices	MPO (%)	TAL		
				x	y	z
Lower right visual quadrant	VBA	1243	55	− 22	− 89	− 2
	SBAV	2074	66	− 26	− 87	2
	CBA	1391	86	− 20	− 93	− 1
Lower left visual quadrant	VBA	853	53	23	− 89	1
	SBAV	1396	56	20	− 93	0
	CBA	918	84	20	− 93	1
Upper left visual quadrant	VBA	958	50	15	− 84	− 13
	SBAV	1401	60	13	− 78	− 16
	CBA	1162	80	18	− 83	− 12
Upper right visual quadrant	VBA	965	47	− 19	− 81	− 14
	SBAV	1897	48	− 14	− 83	− 14
	CBA	1445	76	− 15	− 87	− 15

For each visual quadrant and analysis methods, we counted the number of vertices in the corresponding probability maps exceeding the threshold of 10% probability of activation overlap. For VBA, maximum probability of activation overlap (MPO) was 55%. For SBAV, maximum probability of activation overlap was 66%. For CBA, maximum probability of activation overlap was 86%. We also extracted the Talairach (TAL) coordinates of the vertex showing MPO for each quadrant and each data set.

### Probability difference maps

Probability difference maps (PDMs) revealed a differential impact of the individual methodological elements of our macroanatomical alignment approach.

**Figure 3:**
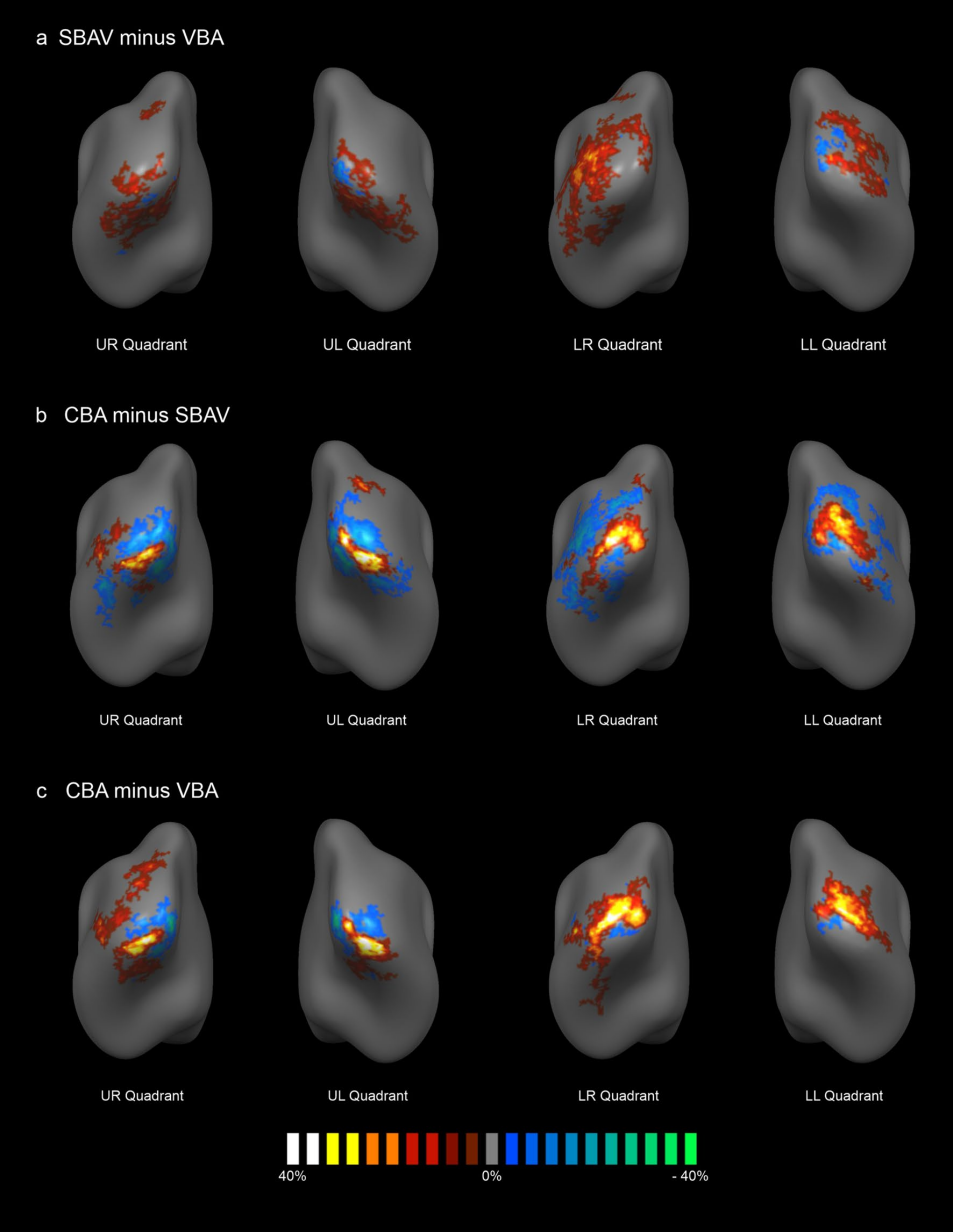
Probability Difference Maps (PDMs). PDMs indicating the differential impact of the individual steps of our overall macroanatomical alignment approach for each visual quadrant. PDMs were generated using PMs derived from single-subject maps. PMs were unthresholded. The color code indicates the difference of activation overlap. The color code brown-to-white indicates a higher degree of functional activation overlap for the more advanced alignment method. The color code blue-to-green indicates a higher degree of functional activation overlap for the less advanced alignment method. PDMs were thresholded at a minimum probability difference of 5%. (**a**) The impact of surface-based functional data readout and pre-processing compared to standard volume-based alignment (SBAV minus VBA) was characterized by a widespread activation with an increase in the probability of activation overlap of up to 30% around the central ROIs and a decrease in the probability of activation overlap of up to 19 % at the location corresponding to the central ROIs. (**b**) The additional impact of macroanatomical alignment (CBA minus SBAV) was less widespread but characterized by an increase in the probability of activation overlap of up to 44% at the location of the central ROIs and a decrease in the probability of activation overlap of up to 32% around the central ROIs. (**c**) The additive impact of both methodological elements (CBA minus VBA) was characterized by an increase in the probability of activation overlap of up to 52% at the location of the central ROIs and a decrease in the probability of activation overlap of up to 36% around the central ROIs.

For pure surface-based functional data readout and pre-processing compared to standard volume-based alignment, the corresponding PDM (SBAV minus VBA) showed a maximum increase in the probability of activation overlap of 30% around the central ROIs. Conversely, at the location corresponding to the central group ROIs we mostly observed a decrease in the probability of activation overlap of up to 19% (Fig. 3a, Table 6). Notably, changes were widespread, partly extending into posterior temporal and parietal cortex. For the addition of macroanatomical alignment, the corresponding PDM (CBA minus SBAV) showed a maximum increase in the probability of activation overlap of 44% in the central ROIs (Fig. 3b, Table 6). Conversely, more peripheral occipital regions showed a maximum decrease in the probability of activation overlap of 32%. Overall, changes were considerably less widespread than for the SBAV minus VBA comparison. For the additive impact of both methodological elements, the corresponding PDM (CBA minus VBA) showed a maximum increase in the probability of activation overlap of 52% in the central ROIs (Fig. 3c, Table 6). Conversely, more peripheral occipital regions as well as posterior temporal and parietal cortex showed a maximum decrease in the probability of activation overlap of 36%. Overall, the spatial extent of these effects fell in between that of the other two comparisons.

**Table 6:** Extent of probability difference maps (PDMs) including positive and negative foci.

Probability difference map	Analysis method	Number of vertices	PD pos (%)	PD neg (%)	TAL PD pos			TAL PD neg		
					**x**	**y**	**z**	**x**	**y**	**z**
Lower right visual quadrant	SBAV minus VBA	1169	30	− 12	− 34	− 76	4	− 16	− 98	− 3
	CBA minus SBAV	1341	40	− 28	− 24	− 90	− 3	− 42	− 75	6
	CBA minus VBA	739	46	− 15	− 25	− 88	− 3	− 24	− 84	9
Lower left visual quadrant	SBAV minus VBA	570	23	− 16	33	− 80	5	20	− 92	10
	CBA minus SBAV	694	38	− 22	22	− 91	5	27	− 77	11
	CBA minus VBA	402	43	− 16	22	− 92	− 2	18	− 96	− 8
Upper left visual quadrant	SBAV minus VBA	612	25	− 19	18	− 72	− 14	12	− 89	− 5
	CBA minus SBAV	680	44	− 32	17	− 82	− 12	10	− 76	− 15
	CBA minus VBA	546	52	− 36	18	− 83	− 12	11	− 86	− 4
Upper right visual quadrant	SBAV minus VBA	689	25	− 13	− 19	− 69	− 9	− 9	− 86	− 7
	CBA minus SBAV	964	36	− 32	− 15	− 87	− 15	− 15	− 72	− 12
	CBA minus VBA	795	43	− 34	− 15	− 87	− 15	− 7	− 84	− 6

For each visual quadrant and comparison between analysis methods (SBAV minus VBA, CBA minus SBAV, CBA minus VBA), we counted the number of vertices in the corresponding PDMs exceeding the threshold of plus 5% or minus 5% difference in probability of activation overlap. Overall, the extent of PDMs was greatest for the CBA minus VBA comparison, i.e., the combined effect of surface-based analysis and macroanatomical alignment. We also extracted Talairach (TAL) coordinates of the positive and negative foci for each quadrant and each data set.

*PD pos* positive value of probability difference, *PD neg* negative value of probability difference.

### Spatial variability of ROI peak vertex distribution (single-subject level)

**Figure 4:**
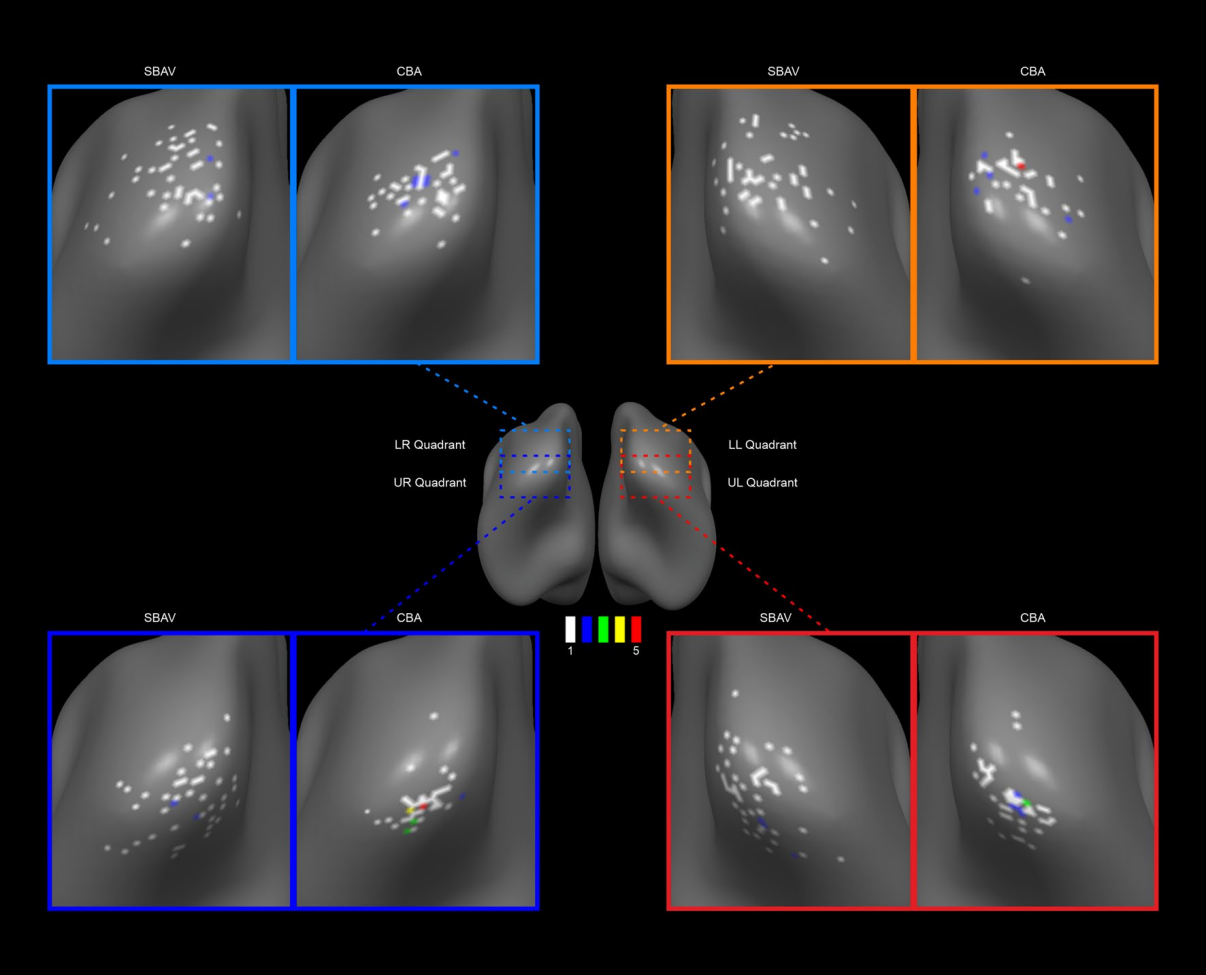
Single-subject peak vertex distribution maps for SBAV and CBA data sets. We mapped single-subject peak vertices for each visual quadrant in surface space for SBAV and CBA data. We then calculated the vertex-wise number of single-subject peak vertices. The color code indicates the number of overlapping single-subject peak vertices per vertex. We observed an increase in the number of overlapping single-subject ROI peak vertices per vertex after macroanatomical alignment (CBA). The number of single-subject peak vertices per occipital vertex for each visual quadrant before and after macroanatomical alignment (SBAV and CBA) ranged between 1 and 5. Thus, a higher number indicates an improved alignment precision of single-subject ROI peak vertices. *LR* lower right visual quadrant, *LL* lower left visual quadrant, *UL* upper left visual quadrant, *UR* upper right visual quadrant.

The rates of success for detecting subject-subject ROIs were as follows: lower right visual quadrant 98% (49 out of 50 subjects), lower left visual quadrant 94% (47 out of 50 subjects), upper left visual quadrant 98% (49 out of 50 subjects), upper right visual quadrant 90% (45 out of 50 subjects). Mirroring group-level PMs, single-subject level peak vertex distribution maps (Fig. 4) showed reduced spatial variability for CBA compared to SBAV. Furthermore, for CBA compared to SBAV we observed an increase in the number of multiple overlapping single-subject ROI peak vertices per vertex for each visual quadrant (Table 7).

**Table 7:** Single-subject ROI peak vertex distribution maps.

# of PV per vertex	LR		LL		UL		UR		Overall	
	SBAV	CBA	SBAV	CBA	SBAV	CBA	SBAV	CBA	SBAV	CBA
1 (no overlapping PV)	45	37	47	34	43	38	41	28	176	137
2 overlapping PV	2	6	0	4	3	4	2	1	7	15
3 overlapping PV	0	0	0	0	0	1	0	2	0	3
4 overlapping PV	0	0	0	0	0	0	0	1	0	1
5 overlapping PV	0	0	0	1	0	0	0	1	0	2
PV detection success rate (%)	98		94		98		90		95	

We mapped all single-subject ROI peak vertices per visual quadrant for SBAV and CBA. We quantified alignment precision of these peak vertices by counting for each occipital vertex the number of overlapping peak vertices for SBAV and CBA. The number of single-subject peak vertices per occipital vertex for each visual quadrant before and after macroanatomical alignment (SBAV and CBA) ranged between 1 and 5. Thus, a higher number indicates improved alignment precision of single-subject ROI peak vertices. After CBA, we observed an increase in the number of multiple, i.e. overlapping, single-subject ROI peak vertices per occipital vertex for each visual quadrant and a corresponding decrease in the number of non-overlapping single-subject ROI peak vertices per occipital vertex. The last row contains the success rate of detecting single-subject ROIs and ergo peak vertices for each visual quadrant. Success rates are necessarily equal for SBAV and CBA, because this aspect of our single-subject analysis cannot be influenced by alignment method.

*PV* single-subject ROI peak vertex, *LR* lower right visual quadrant, *LL* lower left visual quadrant, *UL* upper left visual quadrant, *UR* upper right visual quadrant.

## Discussion

The aim of our study was to evaluate the utility of CBA for an attention-enhanced visual field localizer paradigm used to map circumscribed regions within retinotopically organized visual areas. Our paradigm mapped homologous regions in each visual quadrant reliably across early visual areas. As expected, CBA led to a marked reduction in macroanatomical variability with a number of beneficial effects on the functional level, which clearly exceeded those observed for SBAV. Compared to VBA and SBAV, CBA resulted in the most consistent improvements in the group ROI analysis across visual quadrants (Fig. 1).

For SBAV compared to VBA, we observed a group ROI size increase in three out of four visual quadrants (Fig. 1, Tables 1, 2). For CBA compared to VBA, we observed a group ROI size increase in all four visual quadrants. For CBA compared to SBAV we observed a group ROI size increase in three out of four visual quadrants.

These results indicate an improved power for CBA to detect subregions of early visual areas, which show position selectivity. Conversely, we did not observe an increase of position selectivity within corresponding visual quadrant ROIs across alignment techniques in our linear mixed model analysis (Table 3). However, CBA was the only approach leading to both an increase in ROI size and a preservation not only of vertical but also of horizontal symmetry (Table 4).

Regarding changes in the probability of activation overlap across the three alignment techniques reflected by the PMs, a clear pattern emerged. Probability of activation overlap increased gradually with each step, peaking for CBA with a maximum value of 86%. For SBAV, effects were weaker and considerably more widespread, mostly affecting more peripheral brain regions. Likewise, for the comparison of CBA and SBAV PDMs showed an increase in the probability of activation overlap with a maximum of 44% at the central locations corresponding to the group ROIs. This resulted in considerably more focused activation patterns, while the opposite effect emerged at more peripheral vertices (Fig. 3). This is most likely not attributable to a decreased spatial overlap in the periphery of early visual areas. Rather, it indicates that CBA consistently reduces spurious spread-out activation resulting from poor macroanatomical correspondence after VBA and a generalized SNR increase due to SBAV. It also suggests that VBA and SBAV might misrepresent the location and extent of early visual areas. This notion is supported by changes of the center of gravity of group ROIs between SBAV and CBA, which were particularly pronounced for the lower left visual quadrant (Table 1). Together, these findings indicate that CBA substantially increases statistical power when studying early visual areas at the group level. Naturally, this effect of CBA should also extend to studies with a more global focus, such as connectivity analyses^35,54^.

Additionally, the specific advantages of CBA were evident in the markedly decreased variability of single-subject ROI peak vertex locations for each visual quadrant compared to SBAV (Fig. 4, Table 7). This is indicative of a reduction of macroanatomical and functional inter-subject variability achieved by CBA as the main reason for the improved group-level results. Our findings confirm that transforming functional data from volume-space into surface space already increases statistical power by reducing signal contamination from non-neuronal tissue, thus improving the SNR. Consequently, using SBAV as a proxy for VBA would underestimate the actual benefits of CBA. Our findings indicate that only the CBA approach benefits from both an improved SNR and reduced macroanatomical variability. Thus, our data support the notion that among evaluated methods, CBA is the most advantageous alignment technique for studying the visual system. Such an interpretation is also supported by the fact that only CBA but not SBAV could preserve the vertical symmetry of group ROIs characteristic of early visual areas, which was already evident for VBA (Table 4). This discrepancy is most likely attributable to the unspecific SNR increase induced by SBAV, which in combination with its inherently poor macroanatomical alignment does not result in a consistent improvement of functional overlap for all visual quadrants.

For VBA, we observed the largest group ROIs for the right and left lower visual quadrants, an effect that changed after SBAV and CBA (Fig. 1a, Table 1). For SBAV, we observed the largest group ROIs for the left upper and right lower visual quadrants, which did not persist after CBA (Fig. 1b,c, Table 1). Notably, several studies reported lateralized effects on neurophysiological parameters in early visual areas^55,56^. Our observation raises the question, whether these findings could at least partly be explained by lateralized differences in macro-anatomical variability rather than true functional differences.

Conversely, our CBA-aided group analysis allowed us to compare the response properties of each visual quadrant in a more unbiased way. We observed larger group ROIs for the lower visual hemifield. In a CBA-based probabilistic atlas of the visual system, which included all regions that could be defined in more than 50% of subjects, probabilistic ROIs for dorsal V1 and V2 were also noticeably larger than probabilistic ROIs for ventral V1 and V2, whereas this effect was less clear for V3^43^. These results are in line with our own findings and could be attributable to higher residual anatomical variability after CBA in ventral occipital cortex representing the upper visual hemifield. Alternatively, they could be due to true differences in response properties such as receptive field size or overall area size. The latter interpretation is supported by studies showing functional differences between upper and lower visual hemifields already at the retinal level in the form of differences in receptor densities ^57,58^. Cone density was higher in the superior parts of the retina, which processes information from lower visual fields. Conversely, higher rod density was observed in the inferior parts. Moreover, Eickhoff et al. reported dorso-ventral asymmetries in receptor densities in V2 and V3^57^ and higher GABA-A and muscarinic M3-receptor density in ventral parts of V2 and V3. Furthermore, there is evidence for fundamental differences in receptive field shape from pRF mapping^59^. Estimating both the aspect ratios and the size of mapped areas, a more elliptical receptive field shape was observed for the upper visual hemifield represented by ventral parts of the visual cortex compared to the lower visual hemifield represented by dorsal parts of the visual cortex. Additionally, there is evidence for a behavioral advantage in the lower visual hemifield for shape discrimination as well as higher BOLD-signal changes and peak amplitudes of MEG/EEG responses^50,52,53,60,61^. Together, these findings demonstrate clear differences in the functional architecture of early visual areas representing the upper and lower visual hemifield, respectively. This has been attributed to the fact that the lower visual hemifield represented by dorsal parts of the occipital lobe is more closely linked to the dorsal visual pathway, while the upper visual hemifield represented by ventral parts of the occipital lobe is more closely linked to the ventral visual pathway^62,63^. Furthermore, there is evidence for fundamental differences in receptive field shape from pRF mapping^59^. Here, for the upper visual hemifield represented by ventral parts of the visual cortex, an increased size and more elliptical shape of receptive fields was observed compared to the lower visual hemifield represented by dorsal parts of the visual cortex. This implies that the lower visual field is more specialized for the precise localization and representation of space. Our observation of larger ROIs in the lower visual hemifield is in line with these findings. Hence, our results imply that CBA is a suitable tool to extend the study of functional and behavioral asymmetries in early visual areas to the group-level.

One important limitation of the current study is the lack of complementary retinotopic mapping data due to time constraints. This data would have allowed us to delineate the boundaries of early visual areas and pinpoint the exact visual area containing each individual single-subject ROI. Retinotopic mapping studies indicate that peak activation of single subjects elicited by visual localizers are not consistently located in the same visual area. Most localizer paradigms show peak activation not in V1 but rather in V2 or V3^12^. It is therefore highly likely that our single-subject peak activation did not consistently belong to the same visual cortical area. With the current data set we cannot determine how precisely individual visual areas were aligned with CBA, and whether individual levels of the visual cortical hierarchy were affected differentially. However, the position of our group ROIs, which bordered the calcarine sulcus and spanned the occipital pole, indicate that they mainly comprised V2 and V3. Similarly, after CBA we observed a comparable increase in the probability of overlap in the same part of occipital cortex. While this is at least suggestive of a relatively consistent benefit of CBA across visual areas, more fine-grained studies including retinotopic mapping are required to address this issue more definitively.

Furthermore, we did not use eye tracking to ensure sufficient fixation. We also did not include an additional attentional control task centered on the fixation cross, which would have further encouraged continuous fixation. This omission was deliberate in order to keep the difficulty level adequate for psychiatric patient populations. Our average success rate for finding reliable activation in early visual areas across all four visual quadrants was 95 (90–98)%. Insufficient fixation might partly explain our failure to find reliable activation in a small fraction of subjects.

Finally, several properties of the VBA data set differed from the SBAV and CBA data sets. We could not match volume-based and surface-based pre-processing parameters completely due to inherent differences between the three-dimensional and two-dimensional spatial smoothing algorithms employed. Importantly, the number of voxels and vertices containing functional data were not identical, differentially affecting Bonferroni correction of group results. The smaller analysis space of the VBA data set—69% the size of the SBAV and CBA data set—lead to a corresponding less strict Bonferroni-corrected, final statistical threshold for VBA. Due to this bias towards the VBA data set, the beneficial effects of the additional processing steps featured in the SBAV and CBA data sets should be underestimated. The fact that we could demonstrate the advantages of CBA despite an unfavorable statistical threshold for confirming this primary hypothesis underscores the superiority of this alignment technique.

Our study also has implications beyond mapping the visual system in healthy populations. Visual processing deficits are a prominent feature of neurodevelopmental psychiatric disorders such as ADHD, schizophrenia and autism spectrum disorders^7,8,64–71^, which can also perturb crucial higher-order cognitive processes including working memory^72–74^. The current localizer paradigm will be useful to investigate local impairments of visual information processing as well as disturbances in the interplay between early visual areas and brain networks supporting higher-order cognitive processes. Here, CBA will be particularly relevant to reduce the confounding effects of increased macroanatomical variability in disorders such as schizophrenia in order to measure true group differences and true functional variability^37,75^. On the other hand, CBA might also be crucial for investigating the neurodevelopmental underpinnings of increased macroanatomical variability itself. To this end, the inclusion of probabilistic atlases containing information about gene expression profiles^76^ as well as cyto- and receptor architectonics^77,78^ will be valuable.

Our CBA approach relied solely on cortical curvature information to reduce macroanatomical variability. One main advantage of this method is its feasibility for the vast majority of fMRI data sets, since it only requires a structural brain scan of sufficient quality and resolution. Among comparable methods, CBA is the most data-driven and objective approach. However, the achievable reduction of macroanatomical variability is limited by the variable and imperfect correlation between brain structure and brain function^34,39^. Consequently, more advanced methods additionally utilize orthogonal functional data to further reduce anatomical variability, including the use of functional activation or connectivity patterns to improve macroanatomical alignment across the whole brain^20,79,80^. Additionally, a more complex approach has been proposed, which aligns cortical data using ‘areal features’ more closely tied to cortical areas than cortical folding patterns, including maps of relative myelin content and functional resting state networks^81^. These methods have shown to provide a relevant additional reduction of macroanatomical variability for a variety of paradigms including visual functional localizers. Future studies should also evaluate these methods for retinotopic mapping and visual field localizers. Moreover, it has been demonstrated for early auditory areas, that the additional use of a probabilistic atlas of cytoarchitectonically defined areas further enhances standard CBA results^82^. In principle, such an approach should easily be feasible for the visual system.

To summarize, we demonstrated clear advantages of CBA compared to VBA for the analysis of visual field localizer data at the group-level, signified by a considerable reduction of spatial variability across subjects across early visual areas. Our findings extend previous CBA studies evaluating other major categories of visual mapping techniques. They underscore the loss of information and statistical power incurred by the use of VBA methods in the majority of fMRI studies. Therefore, CBA and comparable methods should be seriously considered as a standard procedure for the detailed study of visual information processing and its disturbance in neuropsychiatric disorders.

## Methods and materials

### Participants

All participants gave their written informed consent to participate in the study in accordance with the study protocol approved by the ethical review board of the Faculty of Medicine at Goethe University. We conducted all experimental procedures in conformity with the approved guidelines and the Declaration of Helsinki. Individuals received compensation for their participation. We recruited 51 healthy volunteers (female:male = 28:23) with age ranging between 18 and 43 years (mean = 24). All participants were non-smokers, had no history of neurological or psychiatric illness and reported normal or corrected-to-normal visual acuity. One participant was left-handed as assessed by the German version of the Edinburgh Handedness Inventory^83^.

### Stimuli and task

**Figure 5:**
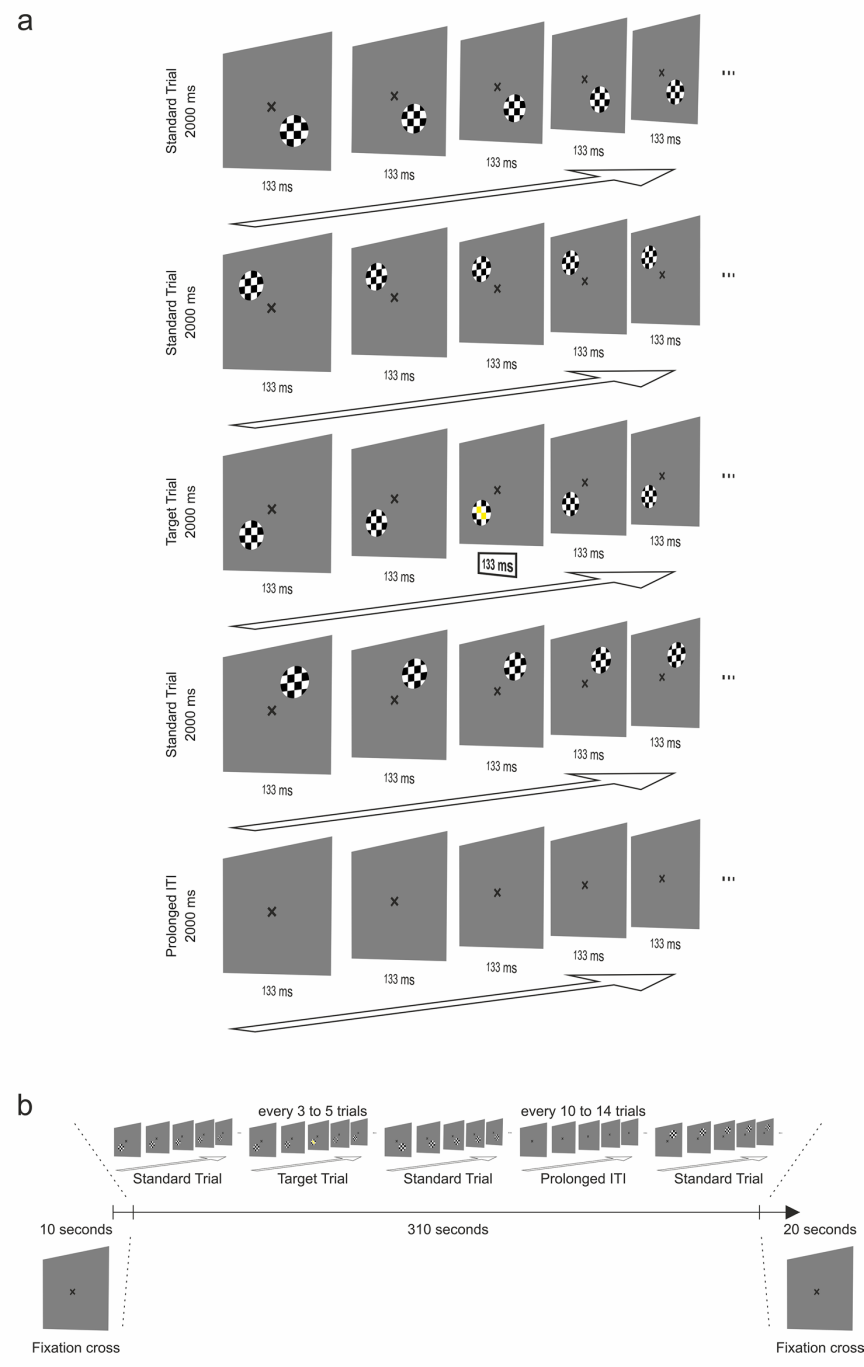
Visual field localizer paradigm. (**a**) The paradigm consisted of flickering, black-and-white colored checkerboards that appeared randomly at homologous positions of the participant’s visual quadrant. In 25% of the trials, the two centrally located squares changed their color to yellow for 133 ms. Participants were required to press a response box button when noticing that. Participants were instructed to continuously fixate a black, x-shaped fixation cross presented at the center of the screen. Checkerboards appeared for 2000 ms. The regular inter-trial interval (ITI) was 0 ms. (**b**) Every 10–14 trials, the ITI extended to 2000 ms. The task comprised 144 trials (25% target trials). It was preceded and followed by a presentation of the fixation cross for 10 s.

Subjects performed an attention-enhanced visual field localizer paradigm (Fig. 5a) implemented using Presentation (Neurobehavioral Systems, Version 18.0) as part of a larger study investigating the role of visual areas for higher cognitive functions. The task consisted of a series of flickering black-and-white-colored round shaped checkerboard stimuli (flicker frequency = 7.5 Hz). Checkerboard stimuli appeared randomly for 2000 ms at one of four different locations (standard trial). Each location mapped a homologous position in one of the four visual quadrants. The regular inter-trial interval (ITI) was 0 ms. However, once every 10–14 trials (11 times overall), the ITI increased to 2000 ms (prolonged ITI) (Fig. 5b). Our paradigm featured a simple target-detection task. During 36 trials, the two centrally located squares of the checkerboard changed their color to yellow for 133 ms (target trial). Participants had to press a response box button with their left thumb as quickly as possible if they detected a target. The paradigm consisted of a total of 144 trials: 36 target trials, 108 standard trials both equally distributed across the four locations (Fig. 5b). This target probability of 25% resulted in one target trial every fourth trial on average (range 3–5 trials) (Fig. 5b). Throughout the task a black, x-shaped fixation cross was displayed at the center of the screen. Participants were instructed to continuously fixate on the fixation cross. Before the first trial, only the fixation cross was displayed for 10 s. After the last trial, only the fixation cross was displayed for 20 s. The total duration of the paradigm was 340 s (Fig. 5b). For the purpose of our analyses we defined a total of four conditions, one for each of the four stimulus locations. Each participant practiced the task prior to the measurement.

### Acquisition and analysis of fMRI data

We acquired functional MRI data on a Siemens 3T MAGNETOM Trio scanner at the Goethe University Brain Imaging Centre using a gradient-echo 2D EPI sequence (32 axial slices, TR = 2000 ms, TE = 30 ms, FA = 90°, FoV = 192 × 192 mm^2^, voxel size = 3 × 3 × 3 mm^3^, gap = 1 mm, effective slice thickness = 4 mm). Slices were positioned parallel to the anterior- and posterior commissure. Functional images were acquired in a single run comprising the acquisition of 170 volumes. Immediately before each functional run, 6 volumes of this 2D EPI sequence were acquired with identical parameters except for a switch of phase encoding direction (posterior to anterior instead of anterior to posterior) for EPI distortion correction. Anatomical MRI data for cortex reconstruction and co-registration with functional MRI data was acquired with a high-resolution T1-weighted 3D volume using a Magnetization-Prepared Rapid Gradient-Echo (MP-RAGE) sequence (192 sagittal slices, TR = 1900 ms, TE = 3.04 ms, TI 900 ms, FA = 9°, FoV = 256 × 256 mm^2^, voxel size = 1 × 1 × 1 mm^3^). Stimulus presentation was constantly synchronized with the fMRI sequence. Head motion was minimized with pillows. The task was projected by a beamer onto a mirror attached on the head coil. MRI data were pre-processed and analyzed using BrainVoyager 20.6^84^, the NeuroElf Matlab toolbox (www.neuroelf.net) and custom software written in Matlab. One subject had to be excluded due to excessive intra-scan motion.

### Structural image pre-processing

Structural data pre-processing included background cleaning, brain extraction and bias field correction to minimize image intensity inhomogeneities^84^. Bias field correction employed a “surface fitting” approach using singular value decomposition based least squares low-order (Legendre) polynomials to model low-frequency variations across 3D image space^85^. We used polynomials with an order of three, which we fitted to a subset of voxels labeled as belonging to white matter. The estimated parameters of the polynomials were used to construct a bias field, which was removed from the data. Our approach comprised of one iteration using automatic white matter labeling^86^ and four iterations using manual white matter labeling.

Subsequently, structural data were transformed into Talairach coordinate space^29^. This comprised manual labeling of the anterior commissure (AC) and posterior commissure (PC) as well as the borders of the cerebrum. These landmarks were then used to rotate each brain in the AC-PC plane followed by piece-wise, linear transformations to fit each brain in the common Talairach “proportional grid” system^19^. Transformation into Talairach coordinate space was performed because the subsequent automatic segmentation procedure exploits anatomical knowledge for initial brain segmentation including removal of subcortical structures and disconnection of cortical hemispheres ^87^. To prepare the data for this procedure, we performed a manual filling of the lateral ventricles. Based on the automatic segmentation of the structural scans along the white–gray matter boundary^87^, cortical hemispheres were reconstructed into folded, topologically correct mesh representations, which were tessellated to produce surface reconstructions and calculate curvature maps reflecting individual cortical folding patterns. Surface reconstructions were subsequently morphed into distortion corrected spherical representations. Finally, both folded and spherical mesh representations were downsampled to a standard number of vertices (40,962 vertices per hemisphere, mean vertex distance: 1.5 mm). We used these standardized mesh representations for all surface-based processing steps.

### Cortex-based alignment of structural data

**Figure 6:**
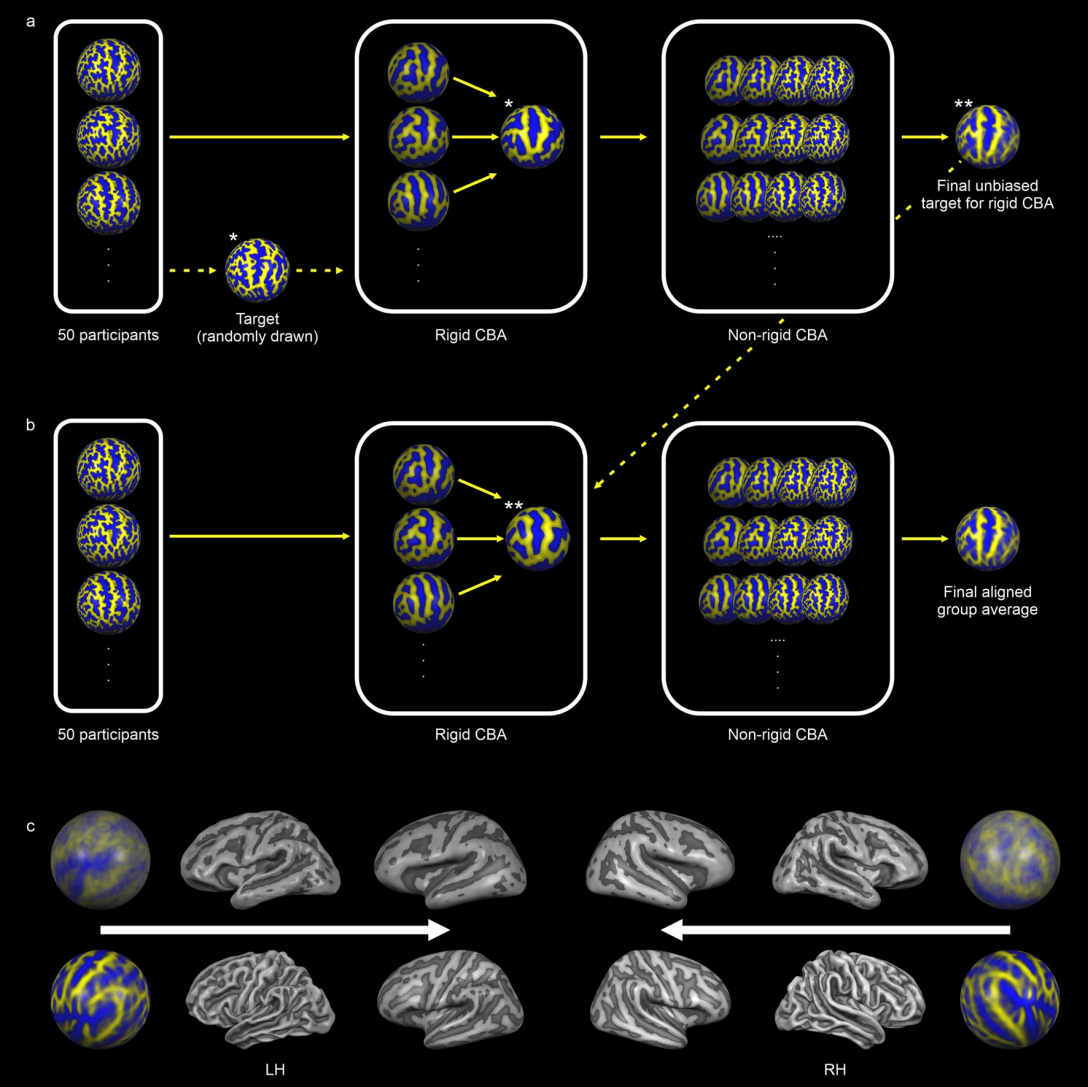
Fully data-driven CBA approach. CBA consisted of a rigid alignment to a single target brain and a non-linear alignment to an iteratively updated group average brain. (**a**) We carried out an initial CBA solely to generate an unbiased average target brain for the final CBA. We used a randomly selected brain from among all participants for the initial rigid CBA. (**b**) For the final CBA we used the unbiased average target brain created during the initial CBA for rigid CBA. (**c**) We generated average surface representations before and after macroanatomical alignment for each hemisphere, which we subsequently merged, inflated and used for analysis and visualization of the appropriate data sets. The upper row depicts group average spherical, folded and inflated mesh representations before applying CBA. The lower row depicts group average spherical, folded and inflated mesh representations after applying CBA.

We then applied a high-resolution, multiscale cortex-based alignment procedure based on the individual curvature maps of all 50 participants for each hemisphere separately. This CBA approach, which reliably aligns corresponding gyri and sulci across subjects^84^, consists of an initial rigid and a subsequent non-rigid alignment step^19^ (Fig. 6a,b). During the initial step, cortical folding patterns of each sphere are aligned rigidly to the cortical folding pattern of a single target sphere by global rotation. Rigid CBA operates solely on highly smoothed curvature maps containing only the most prominent anatomical landmarks. We used the rotation parameters with the highest degree of overlap between the curvature of each individual sphere and the target sphere as the starting point for the subsequent non-rigid CBA.

Non-rigid CBA employs a coarse-to-fine matching strategy, which operates sequentially at four levels of curvature smoothing, starting with the detail level used during rigid CBA. Each subsequent level includes increasingly finer anatomical details up to almost the full curvature information. Importantly, non-rigid CBA aligns each cortical folding pattern to a dynamically updated group average through iterative morphing. This moving target approach, which generates the target curvature map from the average curvature across all hemispheres at a given alignment stage avoids the possible confounding effects of a suboptimal selection of an individual target brain, whose folding pattern might deviate considerably from the cohort average.

Notably, rigid CBA typically utilizes a single brain randomly drawn from the full cohort as its target brain. However, the folding pattern of this brain might also deviate considerably from cohort average. To also address this potential confound, we first conducted a preliminary CBA encompassing both rigid and non-rigid macroanatomical alignment (Fig. 6a). We then conducted a second, final CBA. Here, we used the aligned average brain derived from the preliminary CBA as an unbiased target for the rigid alignment step (Fig. 6b). After the final non-rigid CBA, we merged both hemispheres of each individual brain to create a global surface-based analysis space.

Furthermore, for each hemisphere we created average surface representations from the original, non-aligned folded mesh representations, which we subsequently merged, inflated and used for data analysis and visualization. We repeated these steps after applying the transformation matrix of the final rigid and non-rigid CBA to the folded mesh representations, yielding an accurate representation of the structural effects of macroanatomical alignment (Fig. 6c).

### Functional image pre-processing

**Figure 7:**
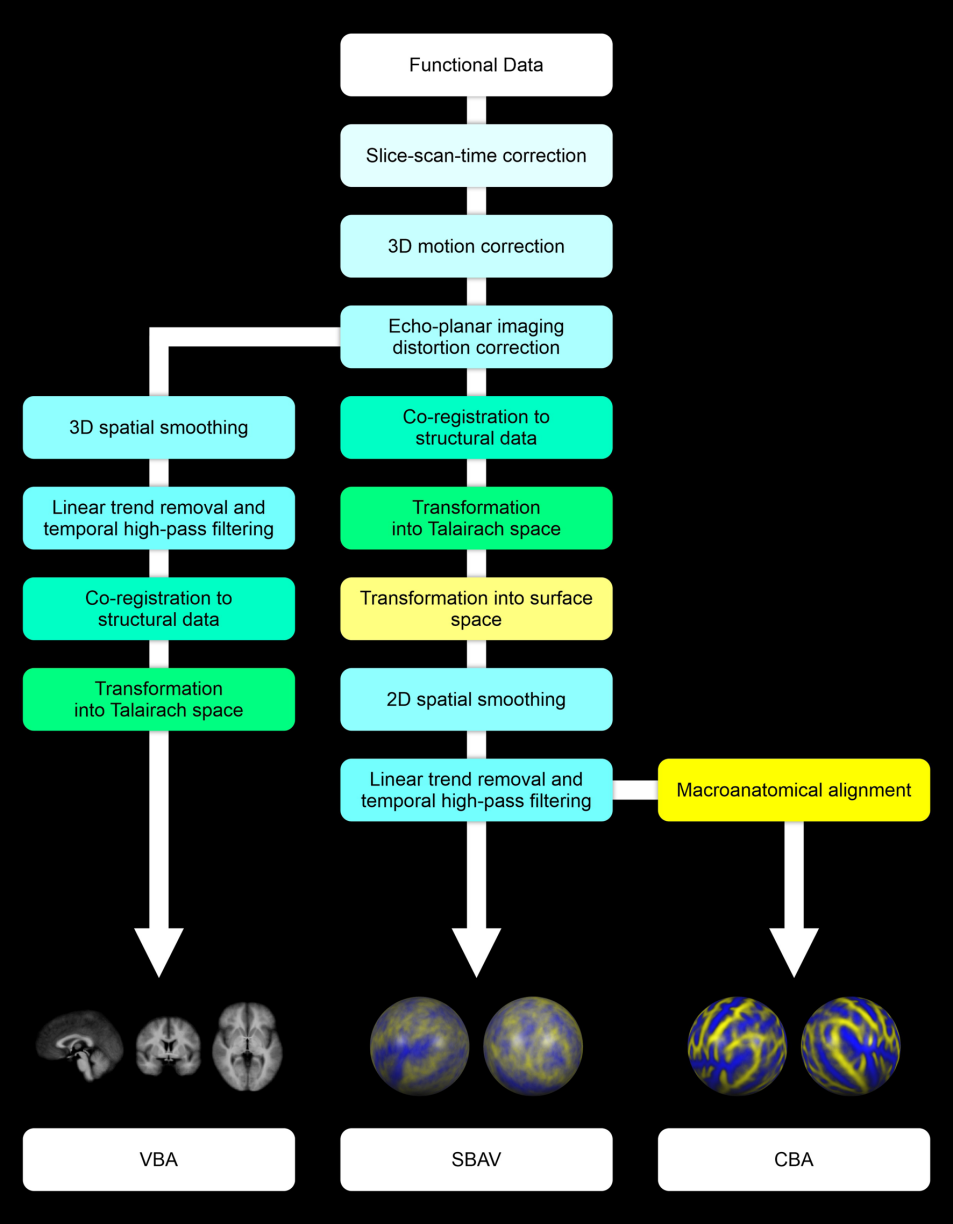
Sequences of functional data pre-processing, coregistration of structural and functional data and spatial transformation operations used to generate the three functional data sets used in our study: VBA, SBAV and CBA. For VBA we conducted all data pre-processing operations in volume space, including slice-scan-time correction, 3D motion correction, echo-planar imaging distortion correction, 3D spatial smoothing and linear trend removal with temporal high-pass filtering. Finally, functional data were co-registered to the structural data and transformed into Talairach space. For SBAV and CBA, we conducted all data pre-processing operations up to echo-planar imaging distortion correction in volume space. Here, co-registration of functional data to the structural data and transformation into Talairach space was followed by transformation into surface space. We then conducted 2D spatial smoothing and linear trend removal with temporal high-pass filtering in surface space. For CBA only, we subsequently applied macroanatomical alignment.

The first four volumes of each functional run were discarded to allow for T1 equilibration. Initial volume-based pre-processing of functional MRI data comprised slice timing correction using sinc interpolation and 3D motion correction using sinc interpolation. Next, we performed echo-planar imaging distortion correction using the Correction based on Opposite Phase Encoding method^88,89^. EPI distortion corrected functional data were co-registered to the untransformed extracted brains. This was accomplished utilizing a boundary-based registration algorithm optimized for surface-based analyses^90^. After co-registration to the fully cleaned but untransformed structural data, functional data were transformed into Talairach coordinate space by applying the transformation matrix generated during Talairach transformation of the structural data using sinc interpolation. This transformation preserved the original voxel size of the functional data (3 × 3 × 3 mm^3^) (Fig. 7).

### Surface-based pre-processing

The volumetric functional data were then transformed into surface space by sampling on the individual cortical surface reconstructions incorporating data from − 1 to + 3 mm along vertex normals using trilinear interpolation. Subsequent pre-processing of fMRI data in surface space started with spatial smoothing using a nearest neighbor interpolation (1 iteration). Based on the standardized vertex distance of 1.5 mm this approximates a 2D Gaussian smoothing kernel with a full width at half maximum (FWHM) of 3 mm. We opted for minimal spatial smoothing to prevent a loss of accuracy for our visual field localizer. Spatial smoothing was followed by linear trend removal and temporal high-pass filtering using fast Fourier transformation (high-pass 0.00903 Hz). Based on the vertex-to-vertex referencing from the folded, topologically correct surface reconstructions to the spherical representations, we mapped the fully pre-processed functional data into a common spherical coordinate system (Fig. 7). Finally, we applied surface-based anatomical masks that only included cortical vertices in our analysis to the functional data. These masks excluded subcortical structures, which mapped onto the midline of our surface reconstructions, i.e., parts of thalamus and the basal ganglia. For functional data analysis and subsequent Bonferroni correction in surface space, this yielded a total number of 76,132 vertices.

### Full volume-based pre-processing

To generate a purely volumetric data set for the comparison of VBA and SBAV, pre-processing after EPI distortion correction was also conducted in volume space mirroring as closely as possible the steps and parameters outlined above for surface-based pre-processing. First, we applied spatial smoothing using a 3D Gaussian smoothing kernel with a FWHM of 3 mm, which approximates the degree of surface-based spatial smoothing. Second, we applied linear trend removal and temporal high-pass filtering using fast Fourier transformation (high-pass 0.00903 Hz) using parameters exactly matching surface-based pre-processing (Fig. 7). This data set was not transformed into surface space and did not include an anatomical mask. For functional data analysis and subsequent Bonferroni correction in volume space, this yielded a total number of 52,504 voxels. Thus, the analysis space for VBA was 69% the size of the analysis space for SBAV and CBA (52,504 voxels vs. 76,132 vertices). This difference lead to a less strict Bonferroni corrected statistical threshold for VBA (p = 0.00000095) compared to SBAV and CBA (p = 0.00000066). Notably, we did not correct for this difference, even though it increased the difficulty of confirming the hypothesized superiority of CBA compared to VBA at the group-level.

### Comparison of functional data sets

Overall, we generated three different functional data sets: a volume-based data set, which was entirely pre-processed and aligned in volume-space (VBA); a surface-based data set, for which the final pre-processing steps—spatial smoothing and temporal filtering—were only applied after transformation in surface space, but without macroanatomical alignment (SBAV); and a surface-based data set, which was pre-processed in exactly the same way as the SBAV data set and also utilized macroanatomical alignment (CBA) (Fig. 7). Accordingly, the primary analysis of these datasets was carried out in volume space (VBA) and surface space (SBAV, CBA) respectively. Planned direct comparisons between these three data sets allowed us to evaluate the effects of different steps of our macroanatomical alignment approach. We compared the VBA and SBAV data sets to assess in isolation the impact of surface-based pre-processing, while keeping macroanatomical alignment constant. We compared the SBAV and CBA data sets to assess in isolation the impact of macroanatomical alignment while keeping pre-processing parameters constant. Finally, we compared the VBA and CBA data sets to assess the combined impact of both surface-based pre-processing and macroanatomical alignment.

### fMRI group analysis of visual quadrants

We performed multi-subject statistical analyses using multiple linear regression of the BOLD signal. The presentation of each checkerboard stimulus sequence at a single location was modelled by an ideal box-car function, which covered the volume of each trial, convolved with a synthetic two-gamma function. These predictors were used to build the design matrix of the experiment. Individual statistical maps were generated by associating each voxel with the beta-value corresponding to the specific set of predictors and calculated on the basis of the least mean squares solution of the general linear model. The resulting individual statistical maps were entered into a second-level random-effects group analysis using a summary statistic approach.

We performed analyses focusing on the mapping of the four visual quadrants at the group level. To define group-level ROIs for each visual quadrant, we computed separate weighted contrasts for each quadrant against the other three quadrants. We assigned a weight of three to the position of interest, e.g. (β_*Quad_1*_ × 3)/(β_*Quad_2*_ + β_*Quad_3*_ + β_*Quad_4*_) (p < 0.05, Bonferroni corrected). This allowed us to detect brain regions showing significant position selectivity. For each resulting group-level ROI, we extracted average time courses (incl. standard errors of the mean) for all four conditions. We conducted this analysis for all three data sets (VBA, SBAV, CBA). For the VBA data set, we computed this analysis fully in volume space using the original resolution of the functional data (voxel size: 3 × 3 × 3 mm^3^). We projected the resulting maps on the non-aligned average surface representation, i.e. the inflated mesh representations before CBA, as depicted in the upper row of Fig. 6c. To this end, volumetric functional maps were transformed into surface space by sampling on the average cortical surface incorporating data from − 1 to + 3 mm along vertex normals of the group average surface brain using trilinear interpolation.

With this transformation we aimed to achieve a visualization and quantification of VBA results equivalent to the SBAV and CBA results. To make all three data sets comparable, this transformation of volumetric functional maps closely mirrored the transformation of functional data into surface space conducted for the SBAV and CBA data sets during pre-processing. Thus, we were able to assess cluster sizes for all ROIs of all data sets in surface space based on the number vertices. We also extracted the number of voxels for the VBA results before transformation into surface space. However, this parameter is not suitable for a comparison with the other data sets and was only included to ensure a comprehensive reporting of our findings.

We used two approaches to determine, whether position selectivity differed between our three data sets: first, to assess differences in the extent of early visual cortex showing significant position selectivity, we compared ROI size, i.e. the number of vertices, for each position of interest across data sets. To this end, we compared quantitative changes in group ROI size between alignment methods utilizing the following formula: {(size_ROI_*Quad*_[AM_*m*_] − size_ROI_*Quad*_[AM_*n*_])/size_ROI_*Quad*_[AM_*n*_]} × 100. Here, Quad indexes the visual quadrant of interest (LR, LL, UL, UR). AM refers to alignment methods (VBA, SBAV, CBA). The subscripted characters n and m specify AMs, with m referring to the less advanced AM and n referring to the comparatively more advanced AM. Accordingly, a positive value indicates an increase in ROI size—and hence position selectivity—for the more advanced alignment method. Second, to test whether the strength of position selectivity within the ROIs of each visual quadrant changed across alignment techniques, we conducted separate linear mixed models with random intercept for each visual quadrant using R (version R 4.1.2). To calculate the degree of position selectivity within each ROI, we contrasted the single-subject t-values of each visual quadrant (“position of interest”) against the single-subject t-values of the three other visual quadrants, e.g. (t_*Quad_1*_ × 3)/(t_*Quad_2*_ + t_*Quad_3*_ + t_*Quad_4*_), separately for each alignment method. We used the results of these contrasts of each subject as the dependent variable and the alignment methods (VBA, SBAV and CBA) as the independent variable. To correct for multiple comparisons, p values were adjusted using Bonferroni correction. Thus, a significant effect in the linear mixed models would indicate a relevant change in position selectivity across alignment methods for a given visual quadrant.

Finally, to assess the impact of the three alignment approaches on horizontal and vertical symmetry of our group-level ROIs, we computed an established asymmetry index (AI)^91^ based on ROI size, i.e. the number of vertices, between each pair of ROIs using the following formula: (|size_*ROI_1*_ – size_*ROI_2*_|/size_*ROI_1*_ + size_*ROI_2*_) × 100. For calculating the vertical AI, we compared the number of vertices of ROIs facing each other at the vertical axis. Thus, for the vertical AI we compared left and right visual quadrants. For calculating the horizontal AI, we compared the number of vertices of ROIs facing each other at the horizontal axis. Thus, for the horizontal AI we compared upper and lower visual quadrants.

### Probability maps

To quantify and visualize variability of functional activation and possible changes induced by macroanatomical alignment, the use of PMs has been proposed. PMs are specifically useful to assess inconsistencies, i.e., disparities between individuals regarding the location of a particular (visual) area^92,93^. To quantify the spatial consistency of position selective activation patterns, we generated PMs for each visual quadrant for all three data sets (VBA, SBAV, CBA). These maps represent the relative number of subjects showing significant task-related activity in our single-subject analysis. To this end, we generated single-subject t-maps based on the same weighted contrasts employed in the group analysis but set at a more lenient statistical threshold (p < 0.05 uncorrected). PMs were calculated by counting the number of subjects showing above-threshold activation in their individual t-maps at a given vertex, dividing this value by the total number of subjects, and multiplying the result by 100. For the VBA data set, we computed all of these steps in volume space and transformed the final PM into surface space using the same parameters outlined above for the volumetric group maps. Finally, we thresholded all PMs at a minimum of 10% probability of activation overlap. We also applied a cluster level threshold of 100 vertices to focus on the main areas of interest, i.e., the visual quadrants. Additionally, we counted the number of vertices in the corresponding probability maps exceeding the threshold of 10% probability of activation overlap for each visual quadrant and analysis methods. Our goal was to quantify and compare the extent of early visual cortex, where each analysis method had a relevant impact on the probability of activation overlap.

### Probability difference maps

Additionally, we aimed to quantify changes in spatial consistency of position selective activation patterns resulting from the different alignment methods. To this end, we calculated PDMs for each visual quadrant, thresholded at a minimum probability difference of 5%, using the original unthresholded PMs. The resulting three PDMs capture different aspects of our overall approach: the impact of surface-based functional data readout and pre-processing compared to volume-based alignment (SBAV minus VBA), the additional impact of applying macroanatomical alignment (CBA minus SBAV) and the additive impact of both methods (CBA minus VBA). Moreover, we counted the number of vertices in the corresponding PDMs exceeding the threshold of plus five or minus five % difference in probability of activation overlap for each visual quadrant. Our goal was to quantify and compare the extent of early visual cortex, where we observed a difference in the probability of activation overlap, for a comparison of analysis method.

### Single-subject ROI peak vertex distribution mapping

For single-subject level analyses, we first defined ROIs for each subject independently before and after macroanatomical alignment, i.e., for SBAV and CBA, using the same weighted contrasts employed in the group analysis. We applied a more lenient statistical threshold (p < 0.05 uncorrected). Next, we determined the peak vertex for each subject’s four visual quadrant ROIs, i.e., the vertex with the highest t-value, for SBAV and CBA. To specifically assess the impact of macroanatomical alignment on the overlap of single-subject ROI peak vertices for each visual quadrant, we mapped all peak vertices per visual quadrant for SBAV and CBA. To quantify changes in the number of precisely overlapping single-subject peak vertices, we counted for each occipital vertex the number of peak vertices for SBAV and CBA. We performed this analysis in addition to the PM- and PDM-analysis to provide a more direct assessment and visualization of the effects of macroanatomical alignment on the spatial correspondence of single-subject ROIs. We restricted this particular analysis to the comparison between SBAV and CBA, because we were specifically interested in studying in isolation the effect of macroanatomical alignment introduced in the CBA data set on the overlap of single-subject ROI peak vertices. Since both VBA and SBAV did not include macroanatomical alignment, but both data sets differed in a number of other pre-processing steps, the direct comparison between SBAV and CBA is the most appropriate to study this particular issue.

## Data Availability

The data that support the findings of this study are available from the corresponding author, R.A.B., upon reasonable request.
